# SARS-CoV-2 Transmission in a Dental Practice in Spain: After the Outbreak

**DOI:** 10.1155/2020/8828616

**Published:** 2020-06-29

**Authors:** Shashi Dadlani

**Affiliations:** Section of Periodontology, Faculty of Medicine and Health Sciences, University Clinic of Odontology University of Oviedo, Oviedo, Spain

## Abstract

The World Health Organization declared a pandemic on March 11, 2020, due to a virus named SARS-CoV-2 discovered in Wuhan, China, in December 2019. Many countries have been hit hard including Spain, with the highest number of healthcare workers being infected (>50,000). A lack of personal protective equipment and protocols at the time of the outbreak led to many fatalities. Although few of these healthcare workers are dental professionals, this community required protective measures as well. Fortunately, there are no reported cases of SARS-CoV-2 transmission in dental practices. Dental professionals were advised only to treat dental emergencies, and such cases were screened via telephone to maintain social distancing. Nevertheless, new protocols and measures are needed as dental professionals return to normal practice after weeks of confinement in many countries. Relatively, few articles have discussed the management of dental practice during the SARS-CoV-2 with no known articles on postpandemic outbreak guidelines. Though some protocols and measures are the same, there are also many differences. Here, we describe protocols and measures for dental practice in Spain in accordance with the Spanish Health Ministry.

## 1. Introduction

A new coronavirus of unknown origin was discovered in Wuhan, China, in December 2019 [1]. It caused an emergent pneumonia outbreak and since then has rapidly spread around the globe. On January 30, 2020, the World Health Organization declared it as a public health emergency of international concern [2], and on 11 February 2020, the virus was given the name SARS-CoV-2 [3].

SARS-CoV-2 is a human-to-human viral infection [4, 5] transmitted through airborne droplets from talking, coughing, or sneezing [6] or by touching or coming into contact with contaminated surfaces that are then transmitted to oral, nasal, and mucosal membranes [7].

This pandemic has severely affected many countries worldwide, causing many deaths in countries such as the USA, UK, Italy, Brazil, France, and Spain [8]. Spain has seen the death of 27,134 people [9]. It has the second highest death rate per 100,000 people in the world after Belgium according to John Hopkins University. Most importantly, more than 50,000 healthcare workers have been infected and are in quarantine; some have tragically died [10]. This high number can be due to the lack of professional protection equipment and protocols in the initial phases of the outbreak. In contrast, Chinese studies showed that only 3.8% of infected people were healthcare workers [11].

Dentists are a low percentage of those affected, and there are no reported cases of SARS-CoV-2 transmission in a dental practice including dental offices; nevertheless, special care is needed in dental facilities. During the coronavirus outbreak, dentists in Spain and other countries were recommended to only attend dental emergencies under strict measures wearing specific professional protection equipment to minimize the risk and maintain social distancing [12]. Routine dental care was also suspended during the outbreak in China [11]. Treatments that could emit aerosol or droplets were postponed or treated without handpieces.

Dental healthcare professionals work in close proximity to patients and therefore have a high risk of being infected [13]. SARS-CoV-2 has been identified in the saliva of infected patients [14], suggesting that the aerosols generated during dental procedures from an infected person can be extremely contagious. These droplets can remain in the area even after the patient has left the clinic, leading to infection of dental professionals via contaminated surfaces [15]. Furthermore, universal precautions must be considered for all patients because asymptomatic patients can also transmit the virus [16].

## 2. Virus Transmission

SARS-CoV-2 is highly transmissible, and there are three ways of viral transmission. Direct transmission is between an infected person and a susceptible individual—this requires contacts such as a handshake. Indirect transmission is via a fomite [14]—an object that has been in contact with an infected person and can thus transmit infection to someone else. Recent studies have shown that SARS-CoV-2 can remain on certain surfaces for up to 9 days [17].

The third mode of transmission is airborne transmission, i.e., via droplets from sneezing or coughing, which can be inhaled by other susceptible individuals [18]. These droplets can be large (>5 *µ*m in diameter) or small (<5 *µ*m in diameter). Large droplets fall to the ground at a faster pace due to gravitational forces, but small ones can stay suspended in the air for a much longer period of time and be inhaled by a susceptible person [19].

## 3. Aerosol Transmission

The media has focused more on direct and indirect transmission of the virus. They emphasize handwashing and not shaking hands with other individuals, especially after touching possible contaminated surfaces; less focus has been given to aerosol transmission.

The airborne spread of SARS-CoV-2 through aerosols in medical procedures has been well documented [14]. Moreover, dental procedures also produce aerosols and droplets that can be contaminated with the virus [18]. The fact that asymptomatic patients can transmit the virus has led us to implement universal protection measures due to cross infection for all patients whether symptomatic or not [16]. It is still unclear exactly how presymptomatic or asymptomatic patients can transmit the virus, but recent literature has confirmed that it is possible [16].

The inhalation of airborne particles and aerosol particles during dental treatments on patients with SARS-CoV-2 is a very high-risk procedure where dentists can be exposed to the virus. Therefore, dental professionals should improve preventive measures to avoid exposure.

## 4. Preventive Measures in Dental Practice after Outbreak

Guidelines on dental care during the SARS-CoV-2 outbreak suggest limiting practice to dental emergencies [11], but it is unclear which protocols should be followed after the outbreak.

Many things will be similar to practice during the outbreak. First, the dental staff should be studied and confirmed to be free of disease with no contact with infected people; ideally, they should be screened daily even after the confirmation of testing negative for SARS-CoV-2. Positive people should be quarantined for 14 days before re-taking the test [20, 21]. Before the patient is given an appointment, telephone screening will be done similar to that done during the pandemic outbreak. Questions will include medical history and symptom characteristics of the virus such as fever, dry cough, sore throat, breathing difficulties, headache, or muscle pain. If any of these symptoms are identified, then treatment should be deferred and sent to a physician [11]. A travel history should also be collected during the last 14 days including any positive contacts. The objective of this screening is to identify a clear rationale for the visit (emergency or just a routine treatment) and to screen the health history.

Patients should come alone to the appointment unless they are a minor, disabled, or elderly people who need help. They should be informed not to wear any jewelry or metal devices to avoid contaminating articles in the dental practice. Punctuality is also very important to minimize waiting room congregation. Patients are also recommended to pay with the credit card to avoid the risk of contaminated bank notes.

Patients should maintain a 2-meter distance with the receptionist. A methacrylate protective screen is recommended to create a barrier between dental staff and the patient. Subjects should not wander around the dental practice unless it is to use the restroom.

All magazines should be removed from the waiting room. Studies have demonstrated that coronavirus can survive 24–30 hours on cardboard and paper [17]. Decoration should also be removed, and patients should ideally be given appointments one at a time to respect social distancing. If this is not possible, then patients should sit 2 meters apart.

Shared areas should be ventilated every 5–10 minutes, and centralized air conditioners should be switched off if they share air from the clinic areas and the shared areas. Negative pressure treatment rooms are recommended for airborne infection isolation rooms including for patients suspected of having SARS-CoV-2 who could be asymptomatic.

SARS-CoV can remain infectious on inanimate surfaces for up to 9 days with greater preference for humid conditions; hence, frequent disinfection of shared areas (door handles, chairs, and washrooms) is mandatory. Surface disinfection with 0.1% sodium hypochlorite or 62–71% ethanol significantly reduces coronavirus infectivity on surfaces within 1 min exposure time. We expect a similar effect against SARS-CoV-2 [17].

Surface disinfection in the dental cabinet should be performed after every patient; the environment must be kept dry. Before entering the dental cabinet, patients should wash their hands with soap for 20 seconds and use hand sanitizers (preferably with 60% alcohol). They should be given disposable surgical shims to cover their shoes. Plastic coverings for dental equipment are needed including the dental chair. The four-handed technique is much more efficient in avoiding cross infection during treatments.

Patients should be given povidone as a mouth rinse because it is highly effective against SARS-CoV and MERS-CoV (related to SARS-CoV-2) [22]. Patients will be advised to rinse their mouth with 0.2% povidone-iodine prior to dental treatment to reduce the viral load in saliva [23]. Other alternatives such as 0.5-1% hydrogen peroxide can also be used but have not yet been shown to have specific viral activity against coronaviruses. Studies with chlorhexidine have not yet been proven to be effective against coronaviruses [15].

During the dental treatment, patients are protected with eye protection, and rubber dam isolation is recommended whenever possible to minimize the production of saliva and blood that could be contaminated. Aerosol emission from handpieces and ultrasonic scalers is restricted during the outbreak.

Extraoral radiographs are advisable because intraoral radiographs could cause a cough reflex. If intraoral radiographs are required, then sensors should be carefully protected to create a barrier and prevent perforation or cross infection [24].

Treatments such as tartrectomies, endodontic access, cavity cleaning, osteotomy surgeries, tooth filing, or implant placement are high-risk procedures due to the aerosols generated. If possible, work at low speed with manual cooling. Other treatments such as manual cleaning, manual scaling, root planning, and dental explorations that do not generate aerosols have a lower risk of infection [25]. High-power aspiration is critical to reduce aerosol production, and the aspirating nozzle should be very near the area that is being treated to avoid aerosol diffusion. Disposable syringes and/or mouth mirrors are recommended to avoid cross contamination. The dental professionals should try to perform the treatment in as few visits as possible [26].

## 5. Professional Protective Equipment (PPE)

PPE minimizes airborne transmission and should be used including impermeable gowns and double gloves [26]. Removing the external glove is recommended after treatment; the internal glove can thus be used to transport material to the sterilization area or to remove any contaminated waste. Thicker gloves are recommended for cleaning.

FFP2 masks are mandatory; they should not have an exhalation valve to avoid diffusion of the virus through exhalation (Spanish Ministry of Health) [27]. FFP2 masks have a 92% particle filtration efficacy and are recommended by the Spanish Health Ministry. No evidence has yet been published proving that FFP3 masks offer better protection from the coronavirus.

These masks can only be used once although Van Straaten et al. suggest that they can be sterilized using hydrogen peroxide vapor in dry heat at 70°C for 30 minutes or humid heat for 15 minutes while retaining function [28].

Eye protection is also critical, and there is strong evidence that the virus can penetrate through the eyes to infect a susceptible individual [29]. Either goggles or protective shields can be used or in some cases both. Finally, impermeable gowns and caps that avoid virus penetration and avoid splatter are recommended [30].

## 6. Conclusion

Dental professionals who are in close proximity to patients are at a high risk of exposure to infection. More data are needed on aerosol transmission in dental practice. Dental professionals have always been prepared to avoid cross infection and have navigated diseases such as HIV and hepatitis; however, SARS-CoV-2 is an airborne disease that is much more contagious. There is still no scientific evidence to accurately conclude when patients are positive but asymptomatic. This makes disease tracing more difficult and transmission more likely. After the SARS-CoV-2 outbreak has flattened, dental colleges and institutions will need new protocols and measures.
